# Real-world uptake of neoadjuvant chemoimmunotherapy and surgery in patients with stage II-IIIB non-small cell lung cancer

**DOI:** 10.3389/fonc.2026.1870140

**Published:** 2026-07-30

**Authors:** Claire Norman, Andrew Giles, Andrew Robinson, Mihaela Mates, Sofia Genta, Maitha Al Sibani, Luxiga Thanabalachandran, Daniel Jafari, Wilma Hopman, Yuchen Li

**Affiliations:** 1 Queen’s School of Medicine, Kingston, ON, Canada; 2 Department of Surgery, Division of Thoracic Surgery, Kingston, ON, Canada; 3 Cancer Centre of Southeastern Ontario, Kingston, ON, Canada; 4 Department of Biomedical and Molecular Sciences, Queen’s University, Kingston, ON, Canada; 5 Clinical Research Centre, Kingston Health Sciences Centre, Kingston, ON, Canada; 6 Department of Public Health Sciences, Queen’s University, Kingston, ON, Canada

**Keywords:** chemotherapy, immunotherapy, neoadjuvant chemoimmunotherapy, non-small cell lung cancer, perioperative therapy, real-world evidence, surgery

## Abstract

**Objective:**

To evaluate real-world uptake, feasibility, and outcomes of neoadjuvant chemoimmunotherapy and surgery among patients with stage II-IIIB non-small cell lung cancer (NSCLC).

**Methods:**

This retrospective cohort study included all patients diagnosed with stage II–IIIB (AJCC 8th edition, excluding N3) NSCLC between August 2022 and August 2024 at the Cancer Centre of Southeastern Ontario (CCSEO). Data on patient characteristics, treatment uptake, surgical outcomes, pathological response, and survival were collected from electronic health records.

**Results:**

Of 587 patients diagnosed with NSCLC during the study period, 132 (22.5%) had stage II–IIIB disease and were included. Thirty-four patients (25.8%) underwent surgical resection, including 10 (29.4%) with upfront surgery and 24 (70.6%) following neoadjuvant chemoimmunotherapy. Overall, 26 patients (19.7%) initiated neoadjuvant chemoimmunotherapy 24/26 (92.3%) completed the planned regimen and proceeded to surgery. Among those undergoing surgery after neoadjuvant chemoimmunotherapy, pathological complete response and major pathological response were observed in 25% and 58.3% of patients, respectively. In exploratory analyses, neoadjuvant chemoimmunotherapy followed by surgery was associated with improved overall survival compared with not receiving this approach (log-rank p=0.009).

**Conclusion:**

In this real-world cohort, fewer than one in five patients with stage II–IIIB NSCLC received neoadjuvant chemoimmunotherapy, highlighting persistent barriers and need for alternative strategies. For those who did receive neoadjuvant chemoimmunotherapy, rates of treatment completion and surgery were higher than those reported in clinical trials..

## Introduction

Lung cancer is the leading cause of cancer-related mortality globally, accounting for approximately one-quarter of all cancer deaths (1). Non-small cell lung cancer (NSCLC) represents the predominant histological subtype. Roughly one-third of NSCLC cases are diagnosed at an early stage, when curative-intent treatments are possible for eligible patients (2). With the growing use of screening and advances, an increasing proportion of NSCLC cases are expected to be identified at earlier stages (3).

For patients with resectable NSCLC, surgical resection remains the cornerstone of treatment. Historically, individuals with large primary tumors 4 cm or larger and/or node-positive disease were recommended to receive up to four cycles of adjuvant chemotherapy to reduce recurrence risk and improve survival (4).

More recently, several phase III trials have investigated neoadjuvant and perioperative systemic approaches combining chemotherapy with immunotherapy for patients with stage IB–IIIA NSCLC. The rationale for neoadjuvant chemoimmunotherapy includes tumor downstaging, the potential to achieve a pathological complete response (pCR), and the opportunity to facilitate more lung-sparing surgery. Collectively, these trials have demonstrated encouraging results, with improvements in both pathological response rates and survival outcomes, supporting the integration of neoadjuvant chemoimmunotherapy into the standard of care for stage II-IIIB NSCLC (5–9).

Nonetheless, despite the clear benefits demonstrated in landmark clinical trials, there remains a paucity of real-world data on the feasibility and effectiveness of neoadjuvant chemoimmunotherapy approaches in routine practice. The primary objective of this study was to evaluate the real-world uptake of neoadjuvant chemoimmunotherapy and surgery among patients with stage II-IIIB NSCLC. In addition, we sought to characterize reasons for not pursuing a neoadjuvant approach and to describe surgical and clinical outcomes in a real-world setting.

## Materials and methods

### Patient population

A retrospective study was completed including patients diagnosed with stage II to IIIB (AJCC 8th edition, excluding N3 disease) NSCLC between August 2022 to August 2024 at the Cancer Centre of Southeastern Ontario (CCSEO). This study was conducted in accordance with the approved protocol by Queen’s University Health Sciences Research Ethics Board (#6042502). The requirement for individual informed consent was waived due to the retrospective nature of the study and use of de-identified data.

### Data extraction and analysis

Patient electronic health records (EHR) were obtained from decision support at Kingston Health Science Centre (KHSC) and used to collect data on patient characteristics, uptake rates of neoadjuvant chemoimmunotherapy and surgery, treatment delays, surgical outcomes, pathological response, and survival information. The data cut-off date for survival and follow-up was June 19, 2025. Follow-up duration was calculated from the date of diagnosis to death or last known follow-up, censored at the data cut-off date. Data were entered into an Excel spreadsheet designed for the study, and imported into IBM SPSS (version 29.0 for Windows, Armonk, New York, 2024) for statistical analysis. Continuous data were described with means and standard deviations, or medians with quartiles as appropriate, and categorical data were described with frequencies and percentages. The underlying distribution of continuous data was assessed with the Shapiro-Wilk test. Factors associated with completion of chemoimmunotherapy with surgery, as well as factors associated with mortality, were assessed using Pearson Chi-square tests, or the Fisher’s Exact test in the event of cell sizes of < 5 cases. T-tests were used for continuous data. Kaplan-Meier curves were utilized to assess survival overall, and to compare survival in patients completing chemoimmunotherapy with surgery in comparison to those that did not. Multivariable logistic regression modelling was used to assess a subset of factors associated with completion, mortality and time to death. Variables were selected for inclusion based on bivariate testing, and clinical relevance. A p-value of <0.05 was used as the cut-point for statistical significance, and no adjustment was made for multiple comparisons.

## Results

### Baseline characteristics

A total of 587 patients were diagnosed with NSCLC during the study period. Of these, 132 had stage II–IIIB disease and were all included in the analysis regardless of surgical, systemic therapy suitability, or comorbidities. At diagnosis, 15 patients (11.4%) had stage IIA, 33 (25.0%) stage IIB, 66 (50.0%) stage IIIA, and 18 (13.6%) stage IIIB disease. Among patients with stage IIIB disease, all had N2 nodal involvement, and half had T3 or T4 tumors by TNM staging (**Table 1A**). Baseline staging for patients with stage II–IIIB disease included positron emission tomography–computed tomography (PET-CT) and magnetic resonance imaging (MRI) of the brain. Mediastinal staging was performed using endobronchial ultrasound (EBUS). The median age at diagnosis was 72 years (IQR: 66–79). Adenocarcinoma was the most common histology (51.5%), followed by squamous cell carcinoma (37.9%) and poorly differentiated subtypes (9.1%). PD-L1 expression was <1% in 21.2% of patients, 1–49% in 28.0%, and ≥50% in 43.9%; EGFR mutations were present in 4.5% of cases, while no ALK alterations were identified. **Table 1B** compared baseline characteristics between patients who received neoadjuvant cheomimmunotherapy and those who did not. A higher proportion of patients who received neoadjuvant chemoimmunotherapy had adenocarcinoma histology and stage IIB compared to those who did not.

**Table 1A:** Baseline patient characteristics.

Characteristic	Overall cohort (N = 132)
Age, years	
Median	72
IQR	66–79
Pathology type	
Adenocarcinoma	68 (51.5)
Squamous cell carcinoma	50 (37.9)
Poorly differentiated	12 (9.1)
Other	2 (1.5)
PD-L1 score	
<1	28 (21.2)
1–49	37 (28.0)
≥50	58 (43.9)
Unknown	9 (6.8)
EGFR mutated	6 (4.5)
ALK mutated	0 (0.0)
Stage at diagnosis	
2a	15 (11.4)
2b	33 (25.0)
3a	66 (50.0)
3b	18 (13.6)

**Table 1B:** Baseline patient characteristics by neoadjuvant chemoimmunotherapy status.

Characteristic	Neoadjuvant chemoimmunotherapy yes (n=26)	Neoadjuvant chemoimmunotherapy no (n=106)
Age, years		
Median	70	73
IQR	65–75	67–79
Pathology type		
Adenocarcinoma	18 (69.2)	50 (47.2)
Squamous cell carcinoma	5 (19.2)	45 (42.5)
Poorly differentiated	2 (7.7)	10 (9.4)
Other	1 (3.8)	1 (0.9)
PD-L1 score		
<1	5 (19.2)	23 (21.7)
1–49	7 (26.9)	30 (28.3)
≥50	13 (50.0)	45 (42.5)
Unknown	1 (3.8)	8 (7.5)
EGFR mutated	0 (0.0)	6 (5.7)
ALK mutated	0 (0.0)	0 (0.0)
Stage at diagnosis		
2a	1 (3.8)	14 (13.2)
2b	11 (42.3)	22 (20.8)
3a	11 (42.3)	55 (51.9)
3b	3 (11.5)	15 (14.2)

### Rate of uptake of surgery and neoadjuvant chemoimmunotherapy

#### Surgery

Overall, 34 of 132 patients (25.8%) underwent surgical resection. Of these 34 patients, 10 patients (29.4%) proceeded directly to upfront surgery, while 24 patients (70.6%) received neoadjuvant chemoimmunotherapy prior to surgery.

Among patients who received neoadjuvant chemoimmunotherapy, only 2 of 26 (7.7%) did not undergo surgery, one due to frailty and severe cognitive impairment identified after treatment initiation, and one due to disease progression while on systemic therapy. Among the 24 patients who underwent surgery after neoadjuvant chemoimmunotherapy, most received either a lobectomy or extended lobectomy (22/24, 91.7%). A minimally invasive approach, video-assisted thoracoscopic surgery (VATS) or robotic was used in two-thirds of cases (66.7%), with the remainder performed via thoracotomy (33.3%). Major postoperative complications (Clavien–Dindo ≥3) occurred in three patients (12.5%), including one postoperative death due to an ILD flare. Recurrence among postoperative survivors was low (12.5%), see **Supplementary Table 1**.

#### Neoadjuvant chemoimmunotherapy

A total of 26 of 132 patients (19.7%) initiated neoadjuvant chemoimmunotherapy, all of whom received at least one dose of chemoimmunotherapy. Most patients (24/26, 92.3%) completed the guideline-recommended number of cycles and proceeded to surgery. Cisplatin-based regimens were used in the majority of patients (61.5%), with carboplatin administered in the remainder (38.5%). Pemetrexed was the most commonly used chemotherapy partner (65.4%), followed by paclitaxel (19.2%) and gemcitabine (15.4%). Nivolumab was the most commonly used immunotherapy agent (80.7%), while the remaining patients received durvalumab (19.2%). The majority tolerated therapy without treatment interruptions (76.9%). No patients with EGFR-mutated disease received neoadjuvant chemoimmunotherapy. Pembrolizumab was not publicly funded for neoadjuvant setting during the study period.

Among the 106 patients who did not receive neoadjuvant chemoimmunotherapy, based on abstraction from clinical documentations, the most common predominant reasons were significant comorbidities, defined as one of more pre-existing medical conditions limiting treatment suitability(36.8%); poor functional status, based on ECOG performance status >=2 and physician discretion (16.0%); anatomical or technical challenges (14.2%), and patient preference, defined as patient refusal or preference not to proceed with neoadjuvant chemoimmunotherapy (12.3%). Unknown was used when no reason was documented (**Table 2**). Of these patients, 11 (8.3%) did not undergo any treatment, 31 (23.5%) received definitive concurrent chemoradiation, 21 (15.9%) received definitive radiation alone (typically 48–60 Gy), and 31 (23.5%) were treated with palliative intent, most with palliative radiation (typically 20 Gy in 5 fractions or 8 Gy in a single fraction).

**Table 2:** Reasons for not pursuing neoadjuvant chemoimmunotherapy.

Reasons for not pursuing neoadjuvant chemoimmunotherapy	Frequency	Percentage (%)
Comorbidities	39	36.8
Poor functional status	17	16.0
Anatomical	15	14.2
Patient declined treatment	13	12.3
Upfront surgery	10	9.4
Unknown	8	7.5
Other	4	3.8

### Predictors of neoadjuvant chemoimmunotherapy uptake

Multivariable analysis identified squamous cell carcinoma as a negative predictor of neoadjuvant chemoimmunotherapy uptake (OR 0.29, 95% CI 0.09–0.92, p=0.04). PD-L1 expression did not influence the likelihood of receiving neoadjuvant chemoimmunotherapy (**Table 3**).

**Table 3:** Factors associated with the likelihood of receiving neoadjuvant chemoimmunotherapy.

	OR	95% C.I. for OR		P-value
		Lower	Upper	
Age at initial diagnosis	0.96	0.91	1.02	0.15
Overall staging				
2a (ref)	-	–	–	0.32
2b	5.79	0.63	53.43	0.12
3a	2.73	0.31	24.41	0.37
3b	3.90	0.32	48.06	0.29
Pathology				
Adenocarcinoma (ref)	–	–		0.10
Squamous cell	0.29	0.09	0.92	0.04
Poorly differentiated	0.42	0.07	2.46	0.34
PD-L1 (%)				
<1 (ref))	–	–	–	1.00
1-49	1.01	0.26	3.91	0.99
>50	0.92	0.27	3.20	0.90
Unknown	0.94	0.08	10.87	0.96
Constant	2.20	–	–	0.74

### Pathological response

Among patients who underwent surgery following neoadjuvant chemoimmunotherapy, pathological complete response (pCR) was achieved in 25% (n=6). The majority of the remaining patients demonstrated a major pathological response (MPR, n=14, 58.3%).

### Survival outcomes

**Figure 1:**
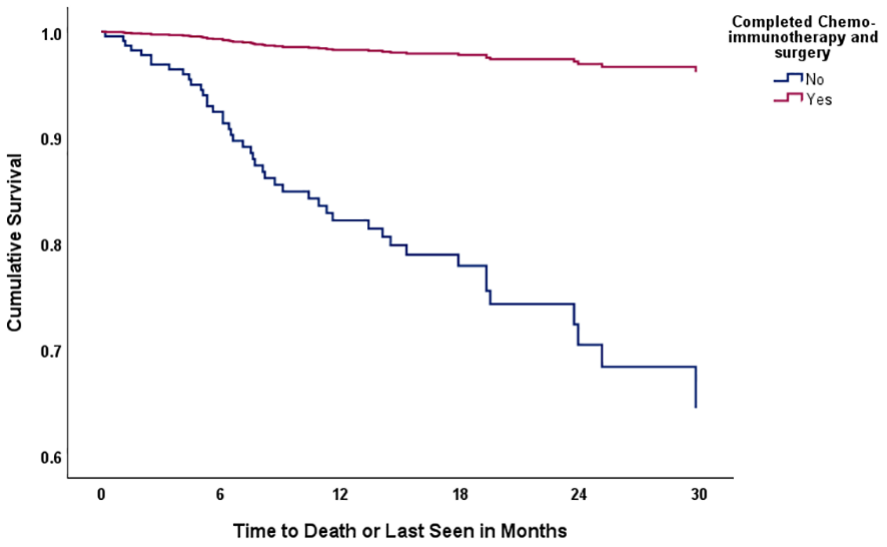
Overall survival in patients completing neoadjuvant chemoimmunotherapy with surgery in comparison to those that did not.

Overall survival was calculated from date of diagnosis to death from any cause of last follow-up. Median follow-up for the overall cohort was 19.0 months (IQR: 12.1–25.2). In this exploratory analysis, receipt of neoadjuvant chemoimmunotherapy followed by surgery was strongly associated with a lower risk of death compared with patients who did not receive this approach (OR 0.08, 95% CI 0.01–0.64, p=0.017; **Table 4**). Kaplan–Meier analysis showed significantly improved overall survival in patients treated with neoadjuvant chemoimmunotherapy and surgery (log-rank p=0.009), see **Figure 1**. Overall, 46 of 132 patients died during follow-up, including 2 of 26 patients (7.7%) who received neoadjuvant chemoimmunotherapy and 44 of 106 patients (41.5%) who did not.

**Table 4:** Patient characteristics associated with mortality.

Variable	OR	95% C.I. for OR	P-value
Completed neoadjuvant chemoimmunotherapy + surgery	0.08	0.01 – 0.64	0.017
Age at initial diagnosis	1.04	0.99 – 1.09	0.168
Overall staging			0.06
2a (ref)	–	–	–
2b	1.18	0.22 – 6.21	0.847
3a	4.63	1.09 – 19.64	0.037
3b	3.83	0.68 – 21.49	0.127
Pathology			0.30
Adenocarcinoma (ref)	–	–	–
Squamous cell carcinoma	2.05	0.82 – 5.09	0.124
Poorly differentiated	4.17	0.75 – 17.38	0.151
PD-L1 (%)			0.64
<1 (ref)	–	–	–
1–49	0.51	0.15 – 1.72	0.275
>50	0.47	0.23 – 2.63	0.418
Unknown	1.00	0.16 – 6.13	0.996
Constant	0.02	–	0.057

**Figure 2:**
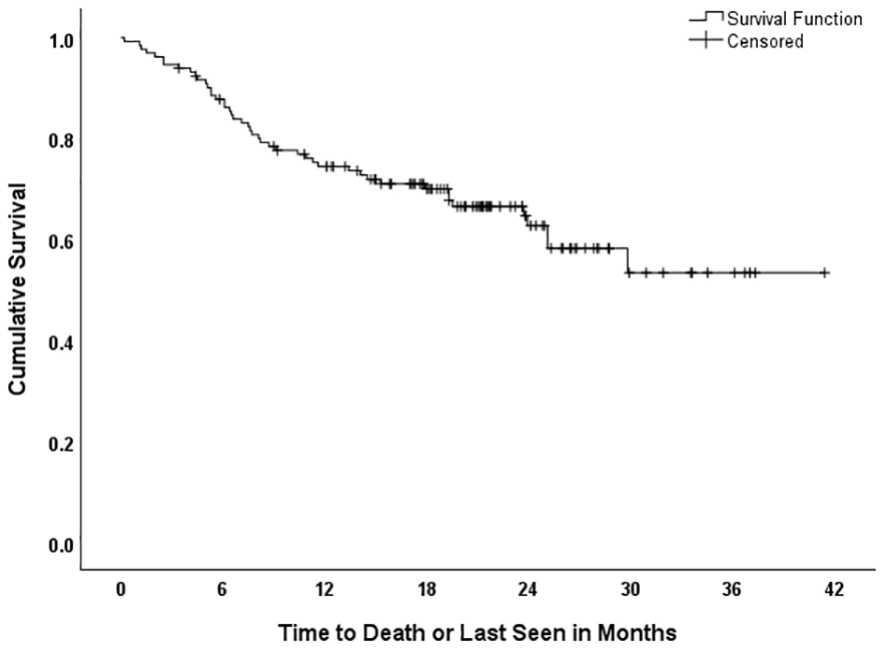
Overall survival over time for the patient cohort.

Among other covariates, stage IIIA disease was significantly associated with worse survival (OR 4.63, p=0.037), while stage IIIB showed a nonsignificant trend toward poorer outcomes (OR 3.83, p=0.127). Squamous histology demonstrated a nonsignificant trend toward increased mortality risk (OR 2.05, p=0.124). PD-L1 expression did not significantly influence survival. Stratified and overall survival curves are shown in **Figures 1** and **2**, respectively.

## Discussion

This study adds to the emerging real-world evidence evaluating the implementation of neoadjuvant chemoimmunotherapy from a population-level denominator within a publicly funded healthcare system. In addition to reporting treatment uptake, our study describes reasons for non-uptake, barriers to implementation, and exploratory insights into the effectiveness of neoadjuvant chemoimmunotherapy in routine clinical practice.

In contrast to current published literature reporting real-world uptake of neoadjuvant treatment in early-stage lung cancer, our cohort included all patients with stage II-IIIB disease. This included those who did not receive neoadjuvant chemoimmunotherapy or who did not proceed to surgery rather than only those receiving neoadjuvant treatment followed by surgery. This approach allowed the quantification of real-world uptake across the broader stage-eligible population and allowed us to show that only a minority of stage II-IIIB patients received neoadjuvant chemoimmunotherapy.

We found that fewer than one-fifth of patients in our cohort received neoadjuvant chemoimmunotherapy, despite its emergence as a new standard of care for stage II-IIIB NSCLC. This low uptake, while notable, is consistent with historical trends. For example, population-based studies in Canada have shown that only approximately one-third of patients received adjuvant chemotherapy when it was previously the standard of care (10, 11). Our study findings also highlight that patients with squamous cell carcinoma were less likely to undergo neoadjuvant chemoimmunotherapy and surgery. This association may be confounded by factors linked to squamous histology, including poorer baseline prognosis, higher smoking exposure, greater comorbidity burden, reduced functional status, and increased risk of treatment-related complications (12, 13).

Importantly, unlike previous clinical trials and reports on neoadjuvant treatment uptake, our study provides real-world data on why most patients were not able to proceed with a neoadjuvant approach. The most common reasons included comorbidities, poor functional status, and anatomical or technical surgical challenges. Advances in surgical techniques and systemic therapy delivery may help broaden eligibility in the future (14, 15). Additionally, a subset of patients (12% in our cohort) declined neoadjuvant chemoimmunotherapy despite no clear contraindications, underscoring the need for improved patient education and navigation to support shared decision-making and increase awareness of the long-term benefits of this strategy. Nonetheless, it is likely that a substantial proportion of patients with stage II-IIIB NSCLC will remain ineligible for neoadjuvant chemoimmunotherapy, highlighting the importance of continued development of alternative treatment strategies.

Among patients who did undergo neoadjuvant chemoimmunotherapy, treatment completion and surgical feasibility were excellent. In our cohort, 92% of patients proceeded to surgery, a rate higher than that reported in pivotal trials such as CheckMate 816 and KEYNOTE-671, where approximately 80% of patients were able to undergo resection after neoadjuvant chemoimmunotherapy (5, 6). This favorable outcome may reflect careful patient selection in our center, as well as the presence of a strong thoracic surgery program with considerable expertise in complex resections. Even when systemic treatment-related complications or delays occurred, surgery was often feasible with good outcomes. Future larger, multi-institutional studies will be important to validate these findings.

In terms of efficacy, the clinical outcomes in our real-world cohort were comparable to those reported in clinical trials. Pathological complete response was achieved in approximately one-quarter of patients. Patients who received neoadjuvant chemoimmunotherapy and underwent surgery also demonstrated improved overall survival compared with those managed without this approach. However, this finding on survival should be interpreted cautiously given the retrospective design, small treated cohort, limited number of outcome events, and potential for immortal time and selection bias. Therefore, the survival findings should be considered exploratory and hypothesis-generating rather than evidence of causal treatment benefit.

This study has several limitations. First, it was a single-institution retrospective analysis with a limited sample size, which restricts generalizability. Second, survival analyses were limited by the relatively few deaths observed, particularly among patients who underwent neoadjuvant chemoimmunotherapy. Third, as with most retrospective studies, there were limitations in case ascertainment: two additional surgical cases captured in the thoracic surgeon’s prospectively-maintained database were not identified in the original decision-support data pull, reflecting known challenges related to coding and data extraction. These cases were not added to avoid introducing selection bias, but their absence may result in conservative estimates of treatment uptake. Next, the study period (2022–2024) preceded the widespread availability of newer neoadjuvant regimens in Canada, such as chemotherapy with pembrolizumab, which may influence current patterns of uptake and outcomes. Finally, because survival was measured from diagnosis and the treatment group was defined by completion of neoadjuvant chemoimmunotherapy followed by surgery, patients in this group necessarily had to remain alive and medically fit long enough to complete treatment and undergo resection. This introduces potential immortal time and selection bias, and survival comparisons should therefore be interpreted as exploratory associations rather than causal treatment effects.

## Conclusion

This study adds to the emerging real-world data assessing the uptake of neoadjuvant chemoimmunotherapy, including reasons for non-uptake and barriers to implementation. Uptake in routine practice was low, with fewer than one in five patients treated, highlighting persistent barriers and the need for alternative strategies. For those who did receive neoadjuvant chemoimmunotherapy, rates of treatment completion and surgery were higher than those reported in clinical trials.

## Data Availability

The raw data supporting the conclusions of this article will be made available by the authors, without undue reservation.
