# Supplementary material for: Real-world uptake of neoadjuvant chemoimmunotherapy and surgery in patients with stage II-IIIB non-small cell lung cancer
Source: Front Oncol. 2026 Jul 30;16:1870140. doi: 10.3389/fonc.2026.1870140 (PMC13467973; doi:10.3389/fonc.2026.1870140)
Supplement: Supplementary file 1 [file Table1.docx]

**Supplementary Table 1. Surgical Characteristics and Post-operative Outcomes Among Patients Undergoing Resection After Neoadjuvant Therapy (n=24)**

| **Patient** | **Extent of Surgery** | **Surgical Approach** | **Major Post-operative Complication** | **Clavien–Dindo Grade** | **Recurrence** |
| --- | --- | --- | --- | --- | --- |
| 1 | Lobectomy | Robotic | None | – | No |
| 2 | Lymph node dissection | VATS | None | – | No |
| 3 | Lobectomy | VATS | None | – | No |
| 4 | Extended lobectomy | Thoracotomy | None | – | No |
| 5 | Lobectomy | VATS | None | – | No |
| 6 | Lobectomy | VATS | None | – | After 3 months |
| 7 | Extended lobectomy | VATS | None | – | No |
| 8 | Extended lobectomy | VATS | None | – | No |
| 9 | Extended lobectomy | VATS | None | – | No |
| 10 | Extended lobectomy | Thoracotomy | Yes | 4A | No |
| 11 | Lobectomy | Thoracotomy | None | – | After 33 months |
| 12 | Extended lobectomy | VATS | None | – | No |
| 13 | Lobectomy | Robotic | None | – | No |
| 14 | Lobectomy | Thoracotomy | None | – | No |
| 15 | Pneumonectomy | Thoracotomy | Yes | 3B | No |
| 16 | Lobectomy | Thoracotomy | None | – | After 3 months |
| 17 | Lobectomy | VATS → Thoracotomy (converted) | Yes | 5 | N/A (deceased) |
| 18 | Lobectomy | VATS | None | – | No |
| 19 | Lobectomy | VATS | None | – | No |
| 20 | Extended lobectomy | Thoracotomy | None | – | No |
| 21 | Lobectomy | Robotic | None | – | No |
| 22 | Lobectomy | VATS | None | – | No |
| 23 | Lobectomy | VATS | None | – | No |
| 24 | Lobectomy | VATS | None | – | No |

*VATs = Video-assisted thoracoscopic surgery
